# The Heart in the Time of the ‘Coronavirus’

**DOI:** 10.5334/gh.786

**Published:** 2020-03-24

**Authors:** Pablo Perel, Diederick E. Grobbee

**Affiliations:** 1 Centre for Global Chronic Conditions, London School of Hygiene and Tropical Medicine, London, UK; 2 Julius Global Health, Julius Center for Health Sciences and Primary Care, University Medical Center Utrecht, NL

**Keywords:** Cardiology, Coronavirus

We are living in extra-ordinary times. The coronavirus disease 19 (COVID-19) pandemic which started in December in China, which has spread around the globe and, as of March 16^th^ 167,511 cases in 152 countries have been reported, and 6,606 related deaths [1,2]. The response is unprecedented and is affecting everyone’s life. It will undoubtedly be a memorable time for many generations.

In parallel to the exponential increase in cases of Covid-19 there is also an *epidemic* of information, data, opinion, and editorials in both medical journals and social media. As of March 16^th^, there are 1,766 papers on the *WHO database on Global Research on COVID-19* published in 2020, which means an average of 23 papers per day [3]. Only on March 11^th^ there were 19 million mentions to COVID-19 in social media [4]. It is indeed very challenging to discern, in this frantic and dynamic situation, the signal from the noise.

There are a few issues for which there is some consensus: the transmissibility and severity is higher than seasonal flu, the pandemic will likely affect every country around the globe, governments need to implement policy and health system country wide actions (although the exact nature of these actions is still a matter of hot debates), and people with pre-existing chronic conditions including cardiovascular disease, hypertension and diabetes appear to be at a higher risk of developing complications and, therefore, at a higher risk of death. As part of the scientific cardiovascular community, we are particularly interested in this latter issue.

Previous studies have shown that people with cardiovascular disease who experienced an acute infection due to other viruses, such as influenza, are at higher risks of cardiovascular events [5]. Also, during previous coronavirus epidemics, such as the one by the Middle East Respiratory Syndrome (MERS), an association between cardiac disease has been described, and specifically there were case reports of MERS related myocarditis [6,7].

The information on the current COVID-19 pandemic is scattered and incomplete. Some studies, mainly from China, have shown that COVID-19 patients have a high prevalence of cardiovascular disease, hypertension and diabetes, but how much this is due to the epidemiological characteristics of the population at risk of COVID-19 (elderly) and how much is due to a specific risk associated with these conditions is not clear [8,9]. Also, some small studies have shown that patients with cardiovascular disease are at a higher risk of complications, such as myocarditis and myocardial infarction, but what are the most frequent cardiovascular complications and which are the patients with cardiovascular disease at a higher risk remain unknown [10]. The information on management strategies for patients with cardiovascular disease and COVID-19 is also limited, one of the most debated topics is the relationship between the angiotensin- converting enzyme 2 (ACE2) and COVID-19. ACE2 has a vital role in the cardiovascular systems and has been also identified as a functional receptor for coronaviruses (infection is triggered by binding of the spike protein of the virus to ACE2) [11]. There have been publications (mainly letters) suggesting a deleterious (or beneficial) effect of ACE inhibitor or angiotensin-receptor blocker on patients with COVID-19, but these are mainly theories [12,13]. Since research is lacking, several international organizations have released statements highlighting the lack of evidence for any harmful effect and asking physicians not to change clinical practice based on this hypothesis [14,15,16].

Current studies are limited in their design (e.g. small numbers, limited geographical representation, lack of data standardization for risk factors and outcomes, limited measurement of confounders, and missing data among other limitations) and therefore is difficult to reach robust conclusions. Some examples of relevant research studies we should be conducting include a better understanding of the cardiovascular conditions that increase the risk of developing COVID-19, better characterization of cardiovascular complications in patients with COVID-19 (e.g. myocarditis), developing simple risk scores to identify patients at high risk of complications to inform triage at the clinical front line, and testing specific treatments and strategies to reduce cardiovascular complications.

Global Heart is therefore inviting researchers across the globe to submit papers related to CVD and COVID-19. We particularly welcome results based on data in low-resource settings.

Also, and in order to help the cardiovascular community to cope with the rapid and emerging evidence we are initiating the *Global Heart & COVID-19 blog* in which we will summarize and comment on the most important articles published on this topic in all journals.

We know it is a very challenging context to conduct research, but we need to try our best to obtain more and better data. In these difficult times, when the foundation of globalization is being challenged, we also need to show the value of collaboration: we encourage sharing protocols, learning from each other and building global collaborations so there are comparable data across the globe.

As part of the cardiovascular community, we are committed to improving the evidence, as much as possible, to inform the care of the hundreds of thousands (if not millions) of people who will be affected by COVID-19.

## References

[1] Thienemann F, Pinto F, Grobbee DE, Boehm M, Bazargani N, Ge J, et al. World Heart Federation Briefing on Prevention: Coronavirus Disease 2019 (COVID-19) in low-income countries. Global Heart. 2020; 15(1): 23 DOI: 10.5334/gh.778 PMC7218761 32489804

[2] WHO. Coronavirus disease 2019 (COVID-19) situation report—56. 3 16, 2020 https://www.who.int/docs/default-source/coronaviruse/situation-reports/20200316-sitrep-56-covid-19.pdf?sfvrsn=9fda7db2_6.

[3] WHO. Global research on coronavirus disease (COVID-19). Database of publications on coronavirus disease (COVID-19). https://www.who.int/emergencies/diseases/novel-coronavirus-2019/global-research-on-novel-coronavirus-2019-ncov.

[4] Managing Brand Communication During Covid-19. Published March 12th at https://blog.sprinklr.com/managing-brand-communication-covid-19/.

[5] Smeeth L, Thomas SL, Hall AJ, Hubbard R, Farrington P, Vallance P. Risk of myocardial infarction and stroke after acute infection or vaccination. N Engl J Med. 2004; 351(25): 2611–8. DOI: 10.1056/NEJMoa041747 15602021

[6] Alsahafi AJ, Cheng AC. The epidemiology of Middle East respiratory syndrome coronavirus in the Kingdom of Saudi Arabia, 2012–2015. Int J Infect Dis. 2016; 45: 1–4. DOI: 10.1016/j.ijid.2016.02.004 26875601 PMC7110824

[7] Alhogbani T. Acute myocarditis associated with novel Middle East respiratory syndrome coronavirus. Ann. Saudi Med. 2016; 36: 78–80. DOI: 10.5144/0256-4947.2016.78 26922692 PMC6074274

[8] Li B, Yang J, Zhao F, et al. Prevalence and impact of cardiovascular metabolic diseases on COVID-19 in China. Clin Res Cardiol. 2020 DOI: 10.1007/s00392-020-01626-9 PMC7087935 32161990

[9] Zhou F, Yu T, Du R, et al. Clinical course and risk factors for mortality of adult inpatients with COVID-19 in Wuhan, China: A retrospective cohort study. Lancet. 2020 https://www.thelancet.com/journals/lancet/article/PIIS0140-6736(20)30566-3/fulltext DOI: 10.1016/S0140-6736(20)30566-3 PMC7270627 32171076

[10] Hui H, Zhang Y, Yang X, Wang X, He B, Li L, Li H, Tian J, Chen Y. Clinical and radiographic features of cardiac injury in patients with 2019 novel coronavirus pneumonia. medRvix. 2020 DOI: 10.1101/2020.02.24.20027052

[11] Zheng Y, Ma Y, Zhang J, et al. COVID-19 and the cardiovascular system. Nat Rev Cardiol. 2020 DOI: 10.1038/s41569-020-0360-5 PMC7095524 32139904

[12] Fang L, Karakiulakis G, Roth M. Are patients with hypertension and diabetes mellitus at increased risk for COVID-19 infection? Lancet Respir Med. 2020 (3 11, 2020). DOI: 10.1016/S2213-2600(20)30116-8 PMC7118626 32171062

[13] https://www.bmj.com/content/368/bmj.m810/rapid-responses.

[14] https://www.escardio.org/Councils/Council-on-Hypertension-(CHT)/News/position-statement-of-the-esc-council-on-hypertension-on-ace-inhibitors-and-ang.

[15] https://ish-world.com/news/a/A-statement-from-the-International-Society-of-Hypertension-on-COVID-19/.

[16] https://www.eshonline.org/spotlights/esh-statement-on-covid-19/.

